# Anxiety and depression among patients with spondyloarthritis

**DOI:** 10.1192/j.eurpsy.2023.1766

**Published:** 2023-07-19

**Authors:** A. Feki, Y. Mejdoub, I. Mnif, I. Sellami, Z. Gassara, S. Bendejemaa, M. Ezzeddine, M. H. Kallel, H. Fourati, R. Akrout, M. L. Masmoudi, S. Yaiich, S. Baklouti

**Affiliations:** 1 Rheumatology; 2 Epidemiology; 3 occupational medecine, Hedi Chalker Hospital, University of Sfax; 4 Rheumatology; 5 occupational medecine, Hedi Chaker Hospital, University of Sfax, Sfax, Tunisia

## Abstract

**Introduction:**

Ankylosing spondylitis (AS) is an inflammatory rheumatic disease characterized by spinal and/or peripheral involvement, enthesitis, dactylitis, and several extra-articular manifestations. Chronic inflammation often leads to reduced spinal mobility and functional disability. The frequency of psychological problems has increased in AS patients.

**Objectives:**

The objective of this study was to determine the prevalence of symptoms of anxiety and depression among AS patients and explore the underlying associated factors.

**Methods:**

The Bath Ankylosing Spondylitis Functional Index (BASFI), the Bath Ankylosing Spondylitis Disease Activity Index (BASDAI), and other clinical measures were collected during the clinical trial. We evaluated also the hospital anxiety and depression scale (HADs). P values < 0.05 were considered statistically significant.

**Results:**

Sixty-two patients with AS were included in the study. The average age was 41 years [18-65]. The diagnostic delay varied from one year to 26 years with an average of 4 years. Twenty-nine years is the average age of onset of symptoms with a standard deviation of 10 years. The mean duration of the disease was 10 ± 8 years. At baseline, the mean BASFI score was 53.9 ± 2 and BASDAI was 4.5± 2.

Clinically significant symptoms of anxiety and depression were present in 48.4% and 54.8% of patients, respectively. Depression was noted with a mean HADS depression of 10,5 ± 5,2. Anxiety was noted with a mean HADS anxiety of 11,3 ± 4,6.

In univariate analysis, anxiety was associated with the low educational level of patients (p = 0.038) and with CRP level (p= 0.041). There was a significant association between depression and anxiety (p=0.000). There was no relationship between these psychiatric disorders and disease activity, treatment modalities or functional status (p>0.05)

**Conclusions:**

In patients with Ankylosing spondylitis, the prevalence of risk of mental disorders is high.

Anxiety and depression are common in AS and even alter the quality of life.

Patients should be regularly screened for these disorders.

**Disclosure of Interest:**

None Declared

